# Salter-Harris Type 1 coracoid process fracture in a rugby playing adolescent

**DOI:** 10.1259/bjrcr.20180011

**Published:** 2018-04-18

**Authors:** George W V Cross, Peter Reilly, Monica Khanna

**Affiliations:** 1 Department of Orthopaedic Surgery, St. Mary’s Hospital, Imperial College NHS Healthcare Trust, London, UK; 2 Department of Radiology, St. Mary’s Hospital, Imperial College NHS Healthcare Trust, London, UK

## Abstract

Fractures of the coracoid process are uncommon and when they do occur, are often mistaken for injuries to the acromi oclavicular joint. We report a case of a 15-year-old boy who sustained a Salter-Harris Type 1 fracture through his coracoid process alongside strain of the acromioclavicular and coracoclavicular ligaments. Additional imaging, specifically MRI, was critical in both correctly identifying this injury as a coracoid process fracture and also in determining that conservative management was the best course of action. Optimum management of such injuries remains controversial, specifically with regards to skeletally immature patients. In our case, the injury was identified clearly on MRI and managed conservatively, with the patient making a full recovery and a return to contact rugby after 3 months.

## INTRODUCTION

Fractures of the coracoid process are uncommon.^1^ When these fractures do occur, they are most frequently associated with other shoulder injuries including: acromioclavicular (AC) joint dislocations, fractures of the scapular spine or acromion, or fractures of the lateral end of the clavicle.^2^ Coracoid process fractures are easily missed and the best management plan is currently under debate. The case presented here is of an isolated coracoid process fracture managed conservatively.

## CASE PRESENTATION

A 15-year-old right-hand dominant schoolboy tackled an opposition player whilst playing rugby. He tackled head-on and, in-so-doing, sustained an impact to the right shoulder. Upon completing the tackle, he was knocked to the ground. There was immediate pain, which he localized to the anterosuperior aspect of the shoulder; this was sufficiently severe to prevent him from finishing the match.

An initial clinical diagnosis of AC injury was made. There was no clinical deformity but tenderness in the region and increased pain when the joint was stressed. The other positive finding of note was that subscapularis testing demonstrated significant pain with preservation of power.

**Figure 1.**
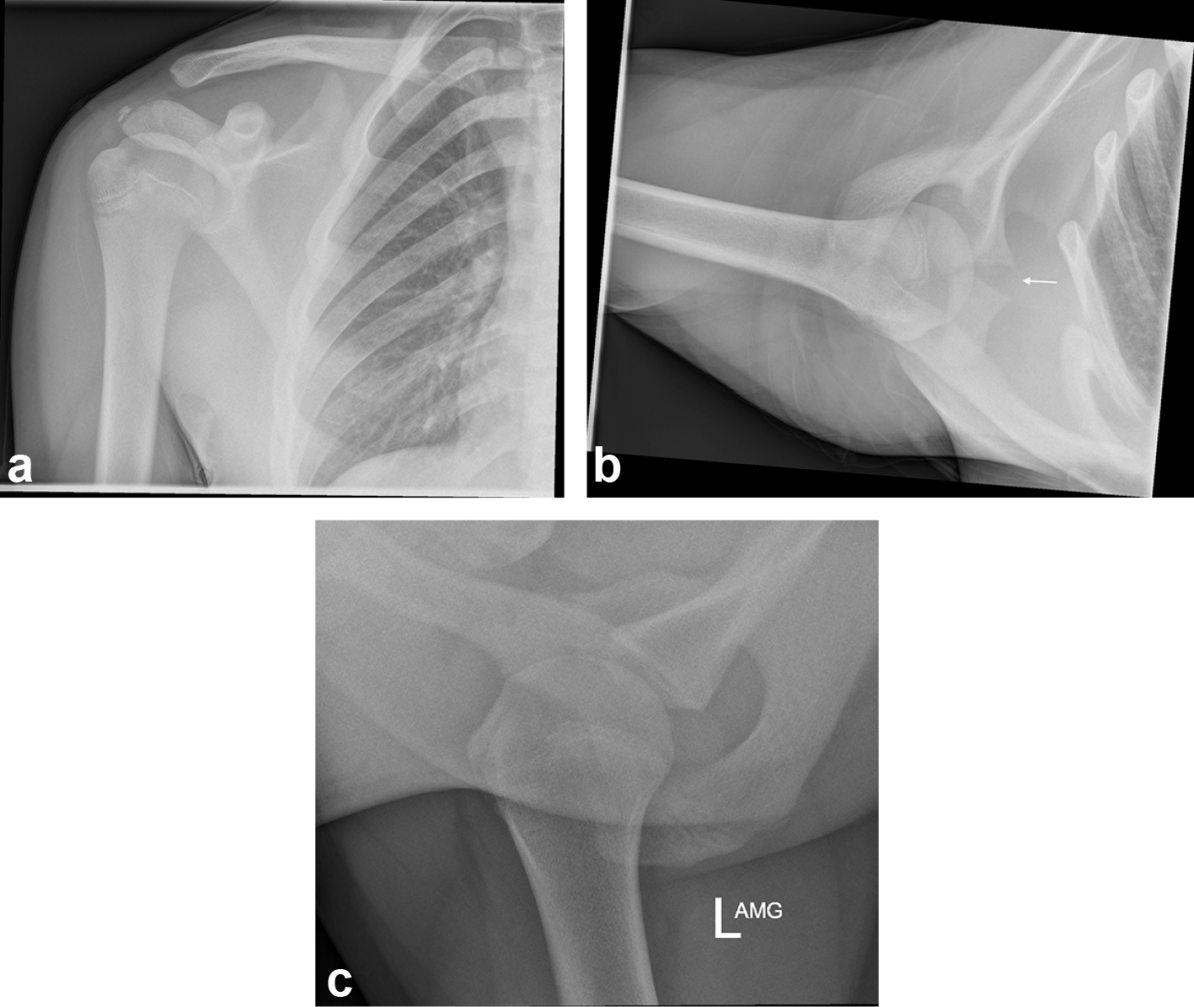
(a) Anteroposterior radiograph of right shoulder showing no definitive bony injury, but with widening of the AC joint. An unfused acromial apophysis is noted. (b) Axial radiograph of right shoulder demonstrates marked widening of the physis at the base of the coracoid process (as shown by the arrow). (c) Axial radiograph of left (normal) shoulder demonstrates normal appearance of an incompletely fused physis at the base of the coracoid process. AC, acromioclavicular.

On the anteroposterior (AP) radiograph, there is apparent widening of the AC joint with superior subluxation of the distal clavicle in relationship to the acromion; with an unfused distal acromial apophysis. The axial radiograph showed normal alignment of the AC joint and marked widening of the physis at the base of the coracoid process (Figure 1a,b). Normal appearance of an incompletely fused physis on the left shoulder further highlights the widening evident on the right side (Figure 1c).

Routine MR shoulder sequences, including a sagittal and axial proton density sequence to give adequate evaluation of hyaline cartilage, were performed 2 days after the aforementioned radiographs.

**Figure 2. f2:**
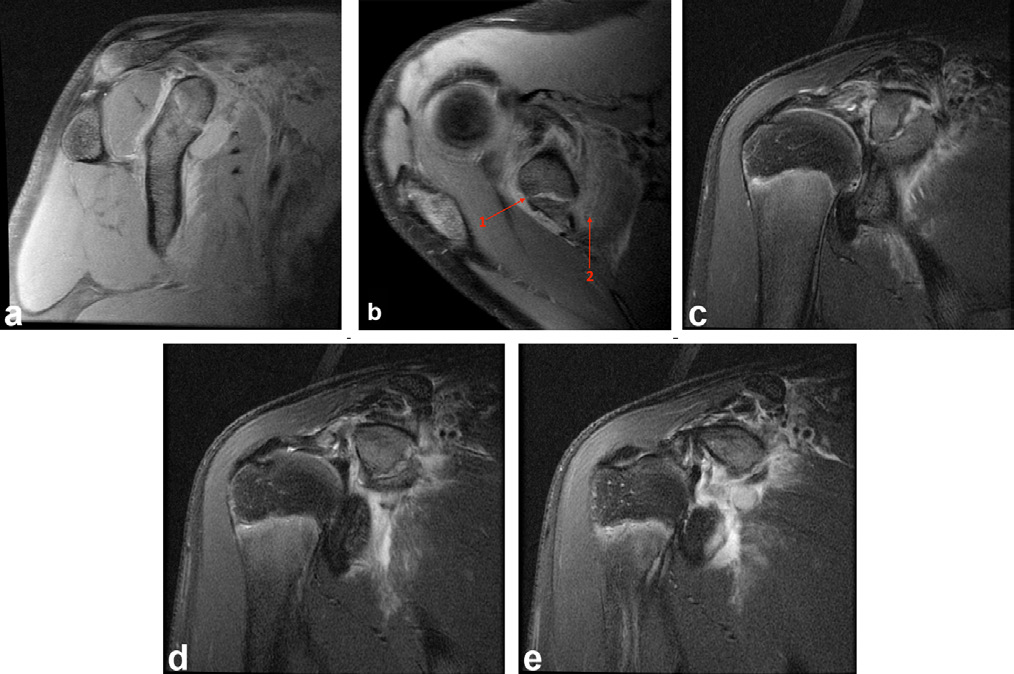
[Fig f2] (a, b) Sagittal and axial Proton Density Fat Saturation (PD FS) sequences demonstrate widening of the physis at the base of the coracoid process with extensive surrounding haematoma and soft tissue oedema at the site of the fracture. *Arrow 1* *,  widening of physis at base of coracoid process;* *Arrow 2,* *surrounding haematoma and soft tissue oedema*. (c*–*e) Coronal *T*_2_ FS sequences demonstrate widening of the physis at the base of the coracoid process with extensive surrounding haematoma and soft tissue oedema at the site of the fracture.

Subsequent MRI confirmed a Salter-Harris Type 1 fracture through the base of the coracoid process with widening of the physis, extensive surrounding haematoma and circumferential soft tissue oedema at the site of the coracoid physeal fracture, extending deep to the supraspinatus and subscapularis muscle bellies, best appreciated on the sagittal view (Figure 2a–e). This patient has a rather unusual vertical configuration of their AC joint on the sagittal sequences, which may furthermore account for the apparent widening at the AC joint on the AP radiograph.

The AC joint demonstrated normal alignment on MR, with a mild strain of the AC ligaments and intermediate strain of the coracoclavicular ligaments.

**Figure 3.**
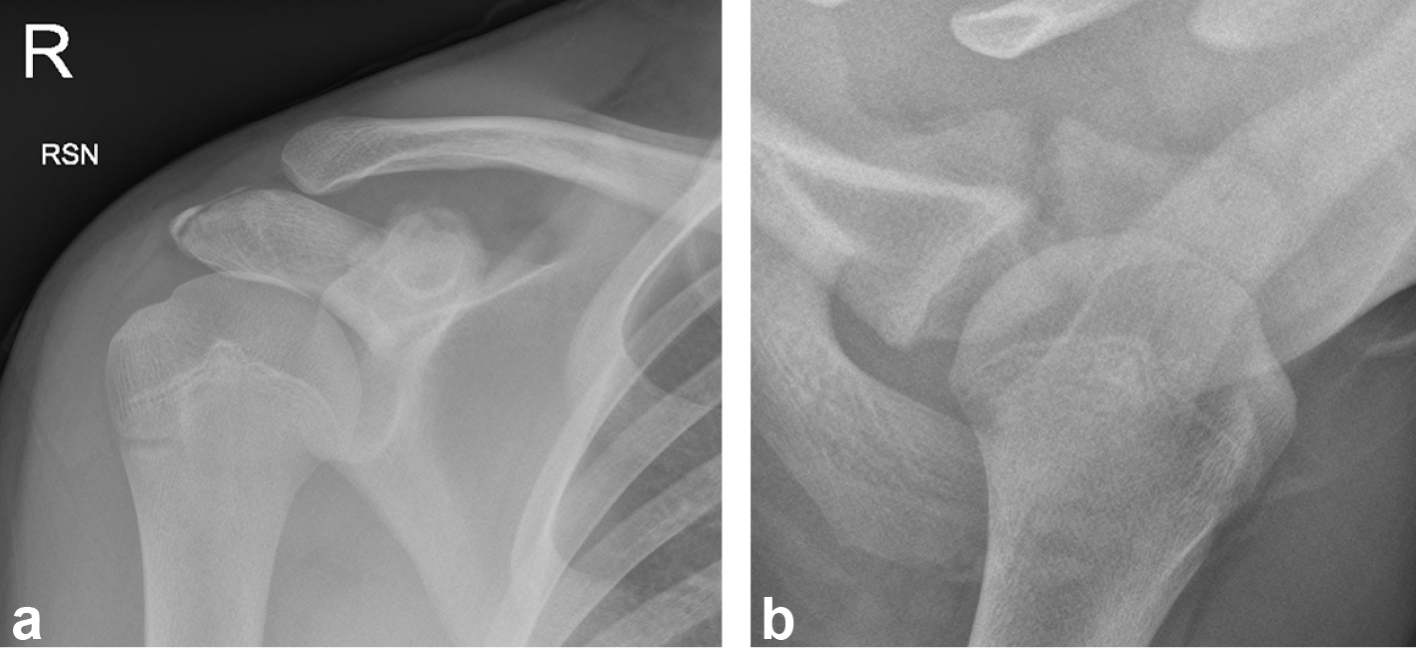
(a) Anteroposterior radiograph of right shoulder showing progressive callus formation at the coracoid physeal injury. (b) Axial radiograph of right shoulder demonstrating the persisting lucency of the physis.

The injury was managed non-operatively with rest in a sling, physiotherapy and a phased return to play. An AP and axial radiograph were taken 10 weeks after the injury with the AP radiograph showing progressive callus formation at the coracoid physeal injury; whilst the lucency of the physis persists on the axial radiograph (Figure 3). The patient fully recovered and returned to playing rugby after 3 months.

## DISCUSSION

Coracoid process fractures are rare, and as such our understanding and knowledge regarding management of these fractures is limited.

**Figure 4.**
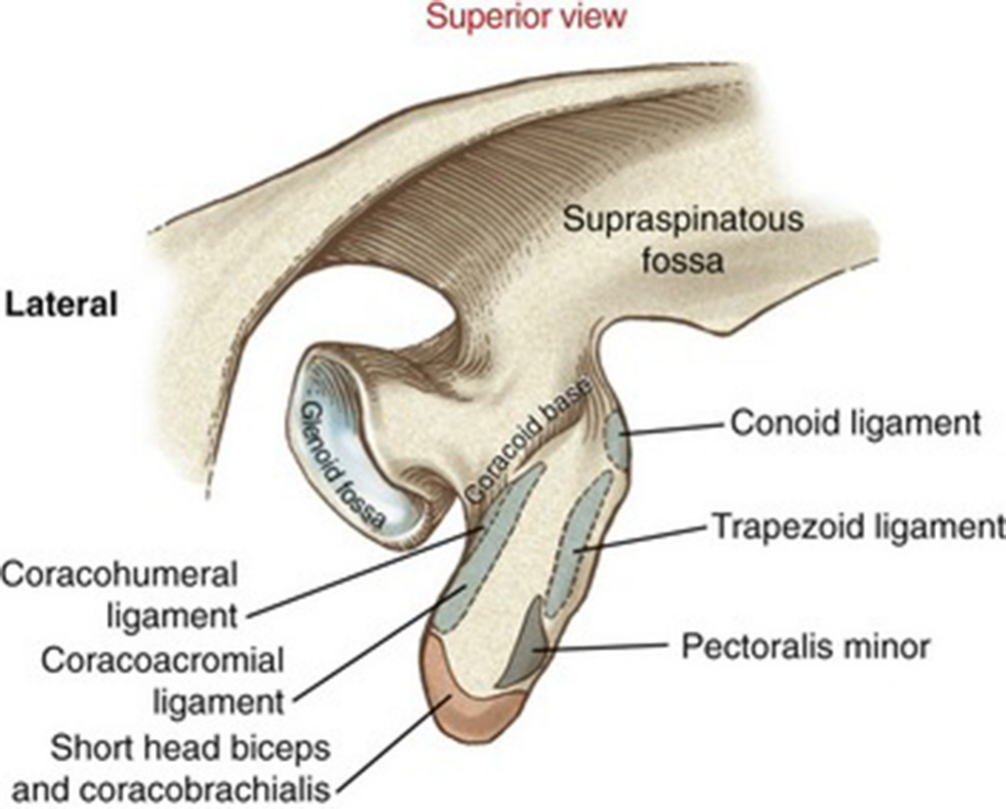
^5^ Superior view of the right coracoid process. Conjoint tendon attachment in red, pectoralis minor attachment in grey. Light blue indicates ligamentous attachments outlined with dashed line. Source: https://clinicalgate.com/shoulder-complex/.

Fracture of the coracoid process appears to occur either through direct trauma, as is the case with this adolescent male patient, or as a result of excessive muscle contraction at the origin of the conjoint tendon.^3, 4^ The corachobrachialis and short head of biceps tendons make up the conjoint tendon, which inserts onto the tip of the coracoid process, with the pectoralis minor tendon being the only other muscular attachment to the coracoid process, inserting along the medial border (Figure 4).^5^

Ogawa et al subdivide coracoid fractures into two types, based on their relation to the coracoclavicular ligaments. Type I fractures are located proximal to these ligaments and are frequently associated with other shoulder injuries. These may require surgical intervention. Type II fractures are located distal to the attachment of the coracoclavicular ligaments and are normally managed conservatively.^2^ Coracoid process fractures are often missed on AP radiographs as the coracoid process is projected on face and physeal displacement may also be minimal. Furthermore, the identification of associated injuries, especially fractures, may lead to satisfaction of diagnosis of the patient’s symptomatology.

It should be mentioned that Ogawa et al did not differentiate between skeletally immature and skeletally mature patients in their subdivision of coracoid process fractures. Skeletal immaturity of a patient adds an additional layer of complexity in identifying coracoid process fractures. A fracture in the skeletally immature group would be difficult to differentiate from a normal unfused secondary ossification centre.^6^ Furthermore, there is variation in ossification timings of both the acromion and the coracoid process, leading to a disparity in the coracoclavicular interval. This finding led Lee et al to propose an alternative method of evaluating and diagnosing AC joint dislocations in the skeletally immature, they suggested placing a greater emphasis on the use of further imaging modalities such as CT.^7^ A further paper by Beranger et al looks at the bone density of the coracoid process in various age groups, and shows a clear decline in density as age increases.^8^ However Beranger et al do not consider the bone density of the coracoid process in a teenage age group, the skeletally immature. Early imaging with MR can aid in establishing an accurate diagnosis of a coracoid physeal injury and allow assessment of associated injuries, which may better inform early management. In the skeletally immature patient, one should have a low threshold for considering a physeal injury, as this is the weakest component in a skeletally immature individual. With evidence to show that coracoid process injuries should be considered in younger athletic patients presenting with shoulder symptomatology.^9^

For our case described above, the Salter-Harris Type 1 fracture satisfies criteria for a Type I coracoid process fracture, set out by Ogawa et al and hence an indication for surgical management. Despite this, a collective decision was made to manage the patient conservatively based on evidence that suggests an early diagnosis of Type I fracture, followed by early implementation of conservative management may still yield functional recovery.^10^ Conservative management is supported in the majority of other reported cases of Salter-Harris Type 1 fractures of the coracoid process, after a subsequent early diagnosis.^11–13^ However, there is a marked difference when applying the conservative management to skeletally mature and immature patients. For the skeletally mature group, the outcomes are mixed with a greater incidence of continuing symptomatology.^14–16^ Amongst the skeletally immature, surgical management, although less frequently applied, has also proven to be more consistently effective, with good post-operative outcomes at 6 weeks following an avulsion fracture.^17 ^ The patients’ skeletal maturity can be used in conjunction with Ogawa et al’s criteria to provide better guidance on the management for such patients, whether surgical or conservative.

Recognizing this unusual injury is important in preventing a misdiagnosis of the more common “AC joint sprain” and an inappropriate early return to contact sport with potentially serious sequelae.

## LEARNING POINTS

1. Coracoid process fractures often go undiagnosed as they tend to be undisplaced and therefore, missed on radiographs.
2. Early imaging with MR is important in establishing an accurate diagnosis and concurrent assessment of associated injuries.
3. Early diagnosis of coracoid process fractures, through MRI, is particularly important in skeletally immature patients, who can benefit from early management options, particularly if conservative.

## Consent

Written informed consent for the case to be published (including images, case history and data) was obtained from the patient(s) for publication of this case report, including accompanying images.
